# Symptom trajectories during chemotherapy in patients with non‐small cell lung cancer (NSCLC) and the function of prolonging low dose dexamethasone in promoting enhanced recovery after chemotherapy

**DOI:** 10.1111/1759-7714.13830

**Published:** 2021-01-25

**Authors:** Jinghao Liu, Xingyu Liu, Ming Dong, Honglin Zhao, Mei Li, Hongbing Zhang, Huihui Ji, Yi Shi, Yajie Cui, Di Wu, Gang Chen, Jun Chen

**Affiliations:** ^1^ Department of Lung Cancer Surgery, Tianjin key laboratory of lung cancer metastasis and tumor microenvironment Tianjin Lung Cancer Institute, Tianjin Medical University General Hospital Tianjin China; ^2^ Department of Thoracic Surgery, First Affiliated Hospital School of Medicine, Shihezi University Shihezi China

**Keywords:** Chemotherapy, low dose dexamethasone, non‐small cell lung cancer (NSCLC), symptom clusters

## Abstract

**Background:**

Lung cancer, mainly non‐small cell lung cancer (NSCLC), is one of the leading causes of death worldwide. Currently, chemotherapy is still the most significant treatment strategy for NSCLC. However, scant attention has been paid in previous studies to those patients who often experience various symptoms and discomfort during chemotherapy treatment cycles.

**Methods:**

This study included 127 NSCLC patients who completed an EORTC QLQ‐C30 questionnaire and specifically designed symptom diary. Chi‐square test, factor analysis, Pearson correlation coefficient and hierarchical cluster analysis were used to perform multivariate analysis.

**Results:**

We identified the top five most‐frequent symptoms within the chemotherapy cycle which included fatigue, insomnia, cough and sputum, appetite loss and hypodipsia. These symptoms were at a moderate level on chemotherapy treatment days 3–7, and were then reduced to a stable and lower level in the following two weeks. A statistically significant difference in adverse events (AEs) was found between 54 patients who received dexamethasone (treatment group) and the control group: fatigue (risk ratio [RR]: 1.48; 95% confidence interval [CI]: 1.120–1.961; *p* = 0.006), insomnia (RR: 1.34; 95% CI: 1.016–1.778; *p* = 0.038), cough and sputum (RR: 2.00; 95% CI: 1.484–2.695; *p* < 0.001), appetite loss (RR: 1.28; 95% CI: 0.959–1.696; *p* = 0.095). In total, 62 patients completed the EORTC QLQ‐C30 scale. The functioning scales of the treatment group were higher than the control population within positive effect sizes (ES: 0.1–0.8). Apart from diarrhea scales, most symptom scales were lower than the control group within negative effect sizes (ES: 0.1–0.9).

**Conclusions:**

In this study we identified the top five most frequent post‐chemotherapy AEs in a chemotherapy treatment cycle and found that dexamethasone was well tolerated by NSCLC patients who received platinum‐based chemotherapy and substantially alleviated the symptom burden and improved the health‐related quality of life (HRQOL) of patients.

## Graphical abstract

In our study, we identified the top five most frequently reported symptoms within a chemotherapy treatment cycle which were fatigue, insomnia, cough and sputum, appetite loss and hypodipsia. After intervention, we found that dexamethasone, with good tolerance, alleviated the symptom burden and improved the health‐related quality of life (HRQOL) of patients.

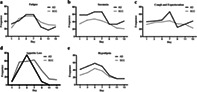

## INTRODUCTION

Lung cancer, mainly NSCLC, is ranked the highest of all cancer‐related deaths worldwide.^1^ The emergence of targeted therapy and immunotherapy has greatly changed the conventional therapy pattern for patients with NSCLC. Limited by selectivity to patients of these two therapies, conventional cytotoxic chemotherapy remains the cornerstone of treatment for NSCLC.^2^ Adjuvant chemotherapy provides great survival benefits, improved overall survival time and significantly progression‐free survival, but patients may suffer from a series of physiological and psychological adverse events (AEs) during treatment with chemotherapy.^3^ This has a direct influence on patients' quality of life (QOL) and functional recovery, with multiple concurrent symptoms often occurring at the same time,^4^, ^5^ and signifies that it is of crucial importance to prioritize relief of these symptoms.

Apart from fatal treatment‐relevant side effects such as severe myelosuppression, what actually causes the most distress to patients are mild but frequently reported symptoms such as fatigue, cough, nausea and vomiting.^6^ These AEs are as a result of both the primary tumor and the cytotoxicity of chemotherapy. Several studies have shown that almost 85% patients of lung cancer suffer from fatigue,^7^ poor quality sleep and insomnia,^8^ and nausea and vomiting are the most frequently reported post‐therapy side effects in patients undergoing chemotherapy.^9^

What deserves our consideration is that these treatment‐related symptoms have been frequently reported to occur in most cases. Two or even more symptoms induced by platinum‐based chemotherapy drugs are often reported to occur at the same time.^3^ These correlative treatment‐related side effects are defined as symptom clusters.^10^ For example, patients may suffer from insomnia, fatigue and depression at the same time. The symptoms of nausea and vomiting may subsequently contribute to the deterioration of anorexia. These symptom clusters are interrelated and interact with each other and have a similar trend of change trajectory.^11^ Under ideal conditions, chemotherapy‐related symptoms should be assessed as symptom clusters due to the relationship between adverse effects, prognosis and QOL.^12^ However, previous studies have usually explored these symptoms individually and only a few studies have previously investigated symptom clusters in cancer patients who have received chemotherapy.^3^, ^13^

Both primary tumor and chemotherapy drugs have been reported to cause AEs in the respiratory, hemopoietic and gastrointestinal systems.^14^ The clinical application of glucocorticoids can alleviate the symptom burden mentioned above. Currently glucocorticoids are the most widely used adjuvant anti‐inflammatory drugs in chemotherapy or radiotherapy for malignant tumors. Glucocorticoids prevent allergic reactions to chemotherapeutic agents, improve digestion and alleviate chemotherapy‐related nausea and vomiting, promote release of mature neutrophils and enhance marrow hemopoietic function, which improves myelosuppression, assists in alleviating cancer pain and also boosts immunity. However, glucocorticoids are double‐edged swords with potential dangers, especially when given at a higher dose as well as being reported to be responsible for a higher incidence of hypertension, insomnia, hyperglycemia, peptic ulcers and so on. However, low doses of glucocorticoids can have a significant effect on the prevention of the side effects of chemotherapy with low cost and great safety.^15^ The most common therapeutic regimen is that patients receive around three days of dexamethasone (about 10 mg, QD) during their perichemotherapy cycle. Nevertheless, a previous study reported the emergence of side effects was focused on the first two weeks in a completed chemotherapy cycle and peaked at an apex level on chemotherapy days 7–9.^13^ The focus of interest in our study was whether prolonging low‐dose dexamethasone promoted enhanced recovery after chemotherapy.

Chemotherapy is still the most important treatment strategy for NSCLC patients at present; however, the obvious side effects caused by chemotherapy dramatically reduce patients' QOL. Currently the development of new anticancer drugs takes up a considerable amount of time of the vast majority of medical investigators. Conversely, what is worthy of consideration is attaching more importance to the terminal care of cancer patients in order to enhance their QOL. Here, a new concept, enhanced recovery after chemotherapy (ERAC), similar to ERAS,^14^ was introduced to summarize our research results. ERAC is meant to reduce the burden of the perichemotherapy symptoms as much as possible with the introduction of some preconditioning methods. Therefore, this study aimed to explore the most common side‐effects which detrimentally affect patients using a symptom diary together with completion of an EORTC QLQ‐C30 questionnaire in order to evaluate their trajectories and interrelation.^16^, ^17^ A further objective of the study was to assess the impact of prolonging the use of low‐dosage dexamethasone on alleviating the symptom burden of chemotherapy.

## METHODS

### Ethical approval and consent to participate

This study was approved by the Ethical Review Committee of Tianjin Medical University General Hospital. Specimens were selected with advance consent of patients and signed informed consent forms. All methods were performed in accordance with the relevant guidelines.

### Sample and settings

This prospective cross‐sectional study used a specific and targeted symptom diary specifically designed by investigators. NSCLC participants were recruited from the Lung Cancer Surgery Department of Tianjin Medical University General Hospital. The recruitment criteria were as follows: (i) patients should be aged 18 years and older; (ii) without cognitive impairment and no other previous diagnosis of malignancy; (iii) diagnosed with NSCLC who underwent standard chemotherapy in the near term; and (iv) the ability to understand and fill out the diary and questionnaire.

After approval by the Ethical Review Committee of Tianjin Medical University General Hospital, 127 patients who met the study criteria were recruited. First, we collected demographic and characteristics in detail from the medical history of the patients. Patients with lung adenocarcinoma received an intravenous injection of D1 of the AP regimen (pemetrexed and platinum drugs) for 2–3 days. Patients with squamous cell carcinoma received D1 of DP (docetaxel and platinum) or the GP (gemcitabine and platinum) regimen. In the GP regimen, gemcitabine was administered on days 1 and 8. Patients in the control group received routine treatment. Patients in the treatment group who received the AP or DP regimen were instructed to continue to take low‐dose dexamethasone (5 mg/QD) for five days after discharge and patients who received the GP regimen continued to receive an intravenous injection of dexamethasone (5 mg/QD) until discharge. As a new chemotherapy cycle commenced, the investigator advised patients of the correlation matters requiring attention in the symptom diary and EORTC QLQ‐C30 questionnaire. The symptom diary had to be completed every two days and the questionnaire completed on the seventh day within the first two‐week of a chemotherapy cycle. Patients were asked to return all paperwork to nurses on returning to the hospital.

A patient undergoing chemotherapy received a series of prophylactic therapy such as acid inhibitors, supportive liver protection therapy and antiemetic therapy. The hemogram index of patients was routinely monitored twice a week. Patients would receive G‐CSFs if their blood cell count decreased, although this situation was rarely encountered.

### Instruments

#### Symptom diary

The symptom diary was specifically designed in order to supervise and urge patients to assess their symptom burden regularly during a chemotherapy cycle which included 18 items such as fever, hiccups, anorexia, appetite loss, depression, dyspnea, hypodipsia, insomnia, ventosity, edema, numbness of limbs/quadriplegia, headache and dizziness, fatigue, cough and sputum, perspiration, pruritus, palpitatations, nausea and vomiting. The diary consisted of two pages, the first included relevant details and announcements, and the second page recorded the symptoms.

#### **EORTC** **QLQ‐C30 questionnaire**

The Quality of Life Questionnaire–core module (EORTC QLQ‐C30) is one of the most widely used cancer‐specific PRO instruments to measure the severity of symptoms and cancer patients QOL. The 30‐item scale comprises five function domains (physical, role, emotional, social and cognitive), three multi‐item symptom domains (fatigue, nausea and vomiting, and pain), six single‐item symptom domains (dyspnea, insomnia, appetite loss, constipation, diarrhea, and financial impact), and two items assessing global QOL domains (the relationship between global and specific components of QOL, assessed with the EORTC QLQ‐C30 in a sample of 2019 cancer patients).

### Statistical analysis

First, we gathered descriptive statistical information about participants' demographic and medical characteristics. A Chi‐square test was used to identify whether the difference of symptom burden between the control and treatment groups had significant interrelation with the application of low‐dose dexamethasone. A factor analysis was conducted to process data from the symptom diary, as well as hierarchical cluster analysis. We then extracted common factors, grouped and clustered different side effects via average linkage between groups, and finally achieved an overall view of patients' chemotherapy‐related symptom burden. Pearson correlation coefficient was used to reflect linear correlation between different symptom burdens. All the statistical computations mentioned above were performed using the Statistical Product and Service Solutions (SPSS 25.0 version).

Furthermore, we also performed specific statistical computations in order to process data from the EORTC QLQ‐C30 questionnaire. We obtained relevant raw scores (RS) by summing up the scores of all items in a function or symptom domain and dividing them by the number of items. Furthermore, we carried out a linear transformation by extreme difference method to transform the RS into standardized score (SS) with a range of 0–100. The calculation formulas are as follows (Table 1):

$$\mathrm{RS}=\left(Q1+Q2+Q3+\ldots \ldots +\mathrm{Qn}\right)/\text{nFunction domains}:\mathrm{SS}=\left[1–\left(\mathrm{RS}–1\right)/R\right]*10\text{0Symptom and global quality of life domains}:\mathrm{SS}=\left[\left(\mathrm{RS}–1\right)/R\right]*100$$

**TABLE 1:** The calculation formulas of EORTC QLQ‐C30

Domain	Abb	No	RS	SS
Physical	PF	5	(Q_1_ + Q_2_ + Q_3_ + Q_4_ + Q_5_)/5	SS = [1–(RS–1)/R]*100
Role	RF	2	(Q_6_ + Q_7_)/2	
Emotional	EF	4	(Q_21_ + Q_22_ + Q_23_ + Q_24_)/4	
Social	SF	2	(Q_26_ + Q_27_)/2	
Cognitive	CF	2	(Q_20_ + Q_25_)/2	
Fatigue	FA	3	(Q_10_ + Q_12_ + Q_18_)/3	SS = [(RS–1)/R]*100
Nausea and vomiting	NV	2	(Q_14_ + Q_15_)/2	
Pain	PA	2	(Q_9_ + Q_19_)/2	
Dyspnea	DY	1	Q_8_	
Insomnia	SL	1	Q_11_	
Appetite loss	AP	1	Q_13_	
Constipation	CO	1	Q_16_	
Diarrhea	DI	1	Q_17_	
Financial impact	FI	1	Q_28_	
Quality of life	QL	1	(Q_29_ + Q_30_)/2	

Abbreviations: Abb, abbreviation; No, number; RS, raw score; SS, standardized score.

## RESULTS

### Patient characteristics

None of the 128 patients (58 females and 70 males) had previously been diagnosed with any other malignancy, and participants' age distribution ranged from 39 to 80 years old. One patient passed away as a result of severe myelosuppression and multiorgan failure after commencing chemotherapy. With regard to the remainder of the participants, 101 patients were diagnosed with lung adenocarcinoma all of whom received several cycles of first‐line platinum‐based chemotherapy such as pemetrexed combined with platinum‐based drugs. A total of 26 patients were diagnosed with lung squamous cell carcinoma who also received a first‐line treatment: gemcitabine/docetaxel and one platinum‐based drug. There were 77 patients without any history of smoking. The clinical stages of 64 patients ranged from I to II, and the rest ranged between III and IV. A total of 98 patients had undergone surgery and an accurate pathological diagnosis had been achieved, and the other 29 patients’ diagnoses had been confirmed by percutaneous lung biopsy and tracheoscopy. The demographic and medical characteristics are shown in Table 2.

**TABLE 2:** Demographic and medical characteristics of patients

	Control group	Treatment group
	*n* = 73	*n* = 54
Gender		
Male	41	28
Female	32	26
Age		
>65	29	21
≤65	44	33
Smoking status		
No	42	33
Yes	31	21
Pathology		
AD	61	40
SCC	12	14
Clinical stage		
I–II	33	31
III–IV	40	23
Metastasis		
No	44	43
Yes	29	11
Lymphatic invasion		
Negative	57	37
Positive	16	17
Adjuvant therapy		
AP	61	40
GP	9	10
DP	3	4
Pathological methods		
Surgery	51	47
Other	22	7

Abbreviations: AD, adenocarcinoma; SCC, squamous cell carcinoma; AP, pemetrexed and platinum; GP, gemcitabine and platinum; DP, docetaxel and platinum.

### Symptom severity and interference

**FIGURE 1:**
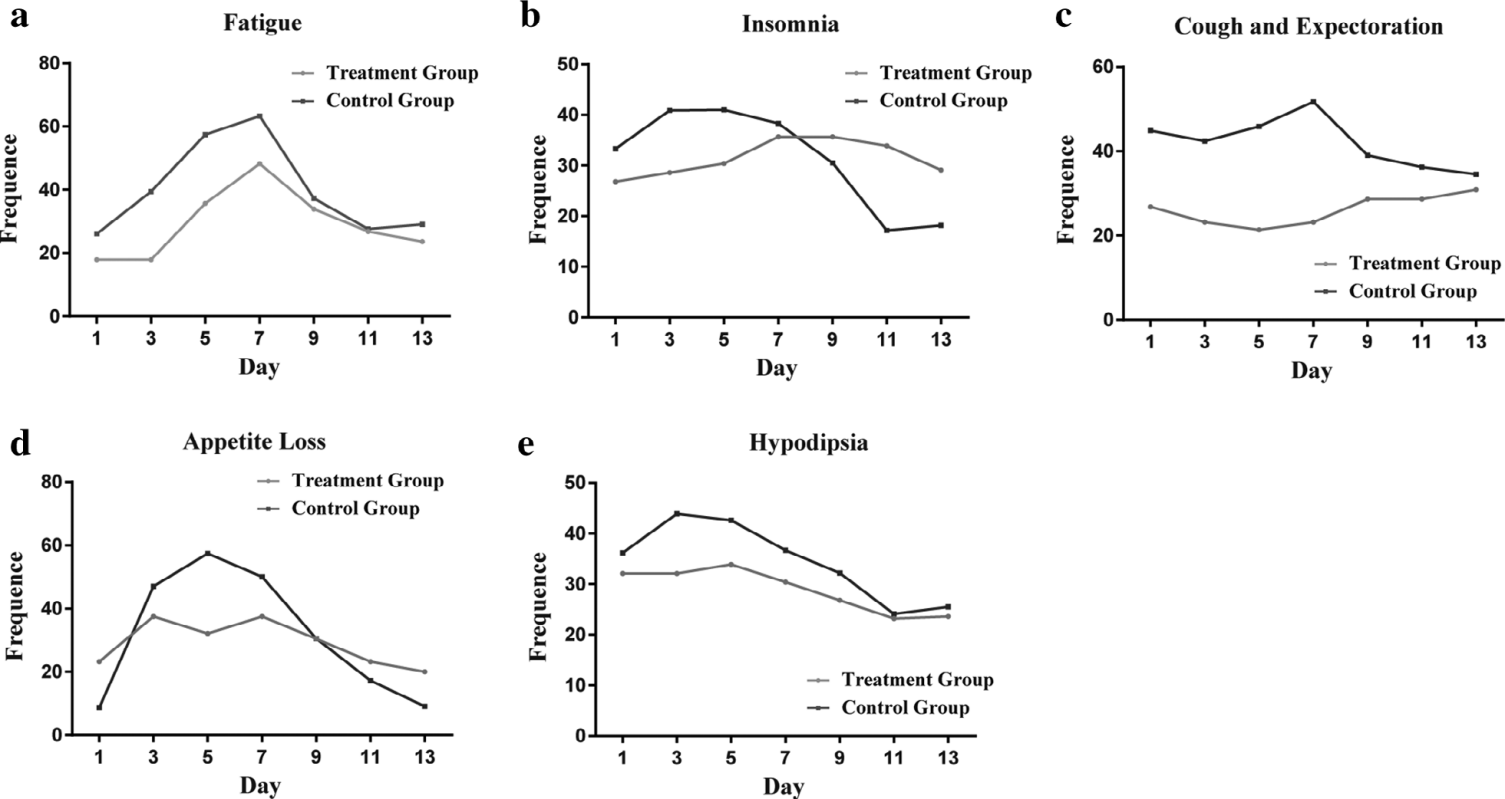
The time‐based symptom appearance rate of the top five most common adverse effects: Fatigue, insomnia, cough/sputum, appetite loss and hypodipsia

The frequency and symptom severity of the common adverse events are shown in Tables 3 and 4. The top five most‐frequent symptoms, in turn, were fatigue (40.5%), insomnia (38.7%), cough/sputum (37.8%), appetite loss (35.2%) and hypodipsia (32.3%). The most severe side effects were insomnia (M, 35.0; SD, 33.0), fatigue (M, 33.5; SD, 19.2), and loss of appetite (M, 21.1; SD, 29.2). These symptoms were at a moderate level on chemotherapy days 3–7, and then reduced to a stable and low level in the following two weeks, as seen in Figure 1.

**TABLE 3:** The frequencies of 18 items from the symptom diary

Symptom	Control group	Treatment group	RR	95% CI		*p*‐value
	Frequency (%)	Frequency (%)		Low	High	
Fatigue	40.5	31.5	1.48	1.120	1.961	0.006
Insomnia	38.7	32.0	1.34	1.016	1.778	0.038
Cough/sputum	37.8	23.3	2.00	1.484	2.695	<0.001
Appetite loss	35.2	29.9	1.28	0.959	1.696	0.095
Hypodipsia	32.3	28.8	1.18	0.881	1.572	0.270
Anorexia	26.8	18.8	1.58	1.146	2.190	0.005
Dyspnea	26.2	21.2	1.32	0.965	1.816	0.081
Nausea/vomiting	25.2	21.2	1.29	1.032	1.615	0.025
Ventosity	23.5	15.6	1.66	1.175	2.343	0.004
Perspiration	20.5	11.6	1.96	1.342	2.872	<0.001
Depression	17.6	15.9	1.13	0.792	1.620	0.494
Headache/dizziness	16.2	9.0	1.96	1.285	2.997	0.002
Palpitations	15.3	7.1	2.34	1.479	3.709	<0.001
Hiccups	8.0	11.4	0.68	0.433	1.066	0.091
Pruritus	6.1	1.1	6.04	2.113	17.257	<0.001
Edema	2.7	4.5	0.60	0.291	1.229	0.158
Numbness	1.8	2.9	0.40	0.145	1.082	0.062
Fever	1.2	2.9	0.60	0.245	1.458	0.253

Abbreviations: CI, confidence interval; RR, risk ratio.

**TABLE 4:** Mean scores and effect size of the treatment and control groups

	Treatment group	Control group			Average of SD	Difference	ES
	M	SD	M	SD			
Functioning scales							
Physical	89.7	9.1	84.8	16.4	12.8	4.9	0.4
Role	92.4	13.7	83.9	24.0	18.8	8.5	0.5
Emotional	92.4	11.5	79.9	20.0	15.7	12.5	0.8
Cognitive	97.0	6.7	86.7	16.3	11.5	10.3	0.9
Social	86.4	29.6	84.4	22.3	26.0	2.0	0.1
Global QoL	77.3	15.4	70.7	20.1	17.8	6.6	0.4
Symptom scales							
Fatigue	18.2	13.4	33.5	19.2	16.3	−15.3	−0.9
Nausea/vomiting	4.5	10.8	7.8	13.8	12.3	−3.3	−0.3
Pain	3.0	6.7	7.2	13.4	10.1	−4.2	−0.4
Dyspnea	9.1	15.6	17.2	21.5	18.5	−8.1	−0.4
Insomnia	21.2	16.8	35.0	33.0	24.9	−13.8	−0.6
Appetite loss	12.1	16.8	21.1	29.2	23.0	−9.0	−0.4
Constipation	6.1	20.1	7.2	15.0	17.6	−1.1	−0.1
Diarrhea	9.1	15.6	3.9	12.3	13.9	5.2	0.4
Financial difficulties	9.1	15.6	16.1	24.7	20.1	−7.0	−0.3

Abbreviations: Difference, difference of mean between the treatment group and control groups; ES, effect size; M, mean; SD, standard deviation.

### Factor analysis and hierarchical cluster analysis

**FIGURE 2:**
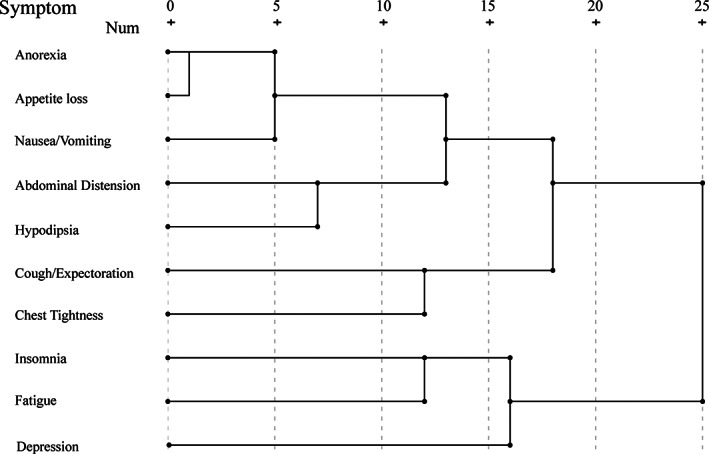
Dendrograms using average linkages. Clusters were formed based on the distances between symptom diary that were calculated using squared Euclidian distances

Factor analysis and hierarchical cluster analysis of the top 10 most frequent symptoms were performed to cluster different symptoms via average linkage between groups in order to obtain homogeneous clusters. Three common factors were identified: fatigue‐insomnia‐emotion cluster, respiratory cluster and gastrointestinal cluster. Relative results of factor analysis are shown in Table 5. The 73 patients in the control group had a greater KMO value which was 0.789 (*p* < 0.001), and a higher KMO meant a stronger relevance between different symptomd and that they were more suitable to perform a factor analysis. The hierarchical diagram is shown in Figure 2 which describes the symptom clusters calculated via squared Euclidian distances. According to the above three common factors, we found three significant clusters in the control group: hypodipsia, anorexia, appetite loss, abdominal distension and nausea/vomiting were gathered as gastrointestinal symptom cluster; fatigue, insomnia and depression were gathered as fatigue‐insomnia‐emotion cluster; cough/expectoration and chest tightness were gathered as respiratory cluster. In conclusion the hierarchical diagram, with obvious aggregation, explained the factor analysis.

**TABLE 5:** Factor analysis with an oblique (oblimin) rotation pattern matrix of symptoms

Symptom cluster	Symptom	Factor loading		
		Factor 1	Factor 2	Factor 3
Gastrointestinal	Appetite loss	0.806		
	Anorexia	0.807		
	Nausea and vomiting	0.802		
	Hypodipsia	0.767		
	Abdominal distension	0.870		
Fatigue‐insomnia‐emotion	Fatigue		0.594	
	Insomnia		0.935	
	Depression		0.766	
Respiratory	Cough and expectoration			0.757
	Chest tightness			0.710
KMO		0.82		
*p*		<0.001		

### Symptom interference

We observed that there had been significant remission in three symptoms compared with the control group, after patients in the treatment group had received a week of low dose dexamethasone treatment (5 mg/QD) (Table 3). The relative risk of fatigue in the control group was 1.48 times higher than in the treatment group (relative risk [RR]: 1.48; 95% CI: 1.120–1.961; *p* = 0.006). The RR of insomnia achieved a 17.3% remission (RR: 1.34; 95% CI: 1.016–1.778; *p* = 0.038), and the RR of cough and expectoration a 38.4% remission (RR: 2.00; 95% CI: 1.484–2.695; *p* < 0.001). As can be seen in Table 4, the health functioning scales of patients were enhanced remarkably within positive effect sizes and all symptom scales achieved significant remission within negative effect sizes, except for diarrhea. In conclusion, low dose dexamethasone was able to help remarkably ease the symptom burden caused by chemotherapy and improve HRQOL of patients.

The results of time series analysis are presented in Table 6 and Figure 1. In general, the symptom development trends of treatment group were parallel and superior to the control group after receiving an intervention of low‐dose dexamethasone. In the control group we observed that the symptom burden of the patients was concentrated around the third to ninth day, and peaked at the fifth day. The development trend then decreased to a stable and lower level in the remainder of the first two weeks. It is significant that the symptom development trends of the treatment group were below the control group in the tendency chart (Figure 1) which presented the trends of the five most common symptoms. What we elucidated from Table 6 was that the symptom burden of side effects was significantly relieved on the third and fifth days (*p* < 0.05).

**TABLE 6:** The results of time series analysis and difference between the treatment and control groups

Time	Symptom									
	Fatigue		Insomnia		Cough and sputum		Appetite loss		Hypodipsia	
	RR	*p*	RR	*p*	RR	*p*	RR	*p*	RR	*p*
D1	0.616	0.272	0.732	0.429	0.448	0.036	3.174	0.025	0.834	0.632
D3	0.360	0.015	0.622	0.217	0.443	0.040	0.735	0.402	0.653	0.256
D5	0.540	0.093	0.767	0.489	0.399	0.022	0.460	0.037	0.849	0.664
D7	0.760	0.445	1.111	0.781	0.371	0.011	0.780	0.499	0.872	0.725
D9	1.097	0.809	1.574	0.245	0.800	0.568	1.235	0.597	0.963	0.925
D11	1.212	0.643	3.030	0.010	0.914	0.820	1.784	0.211	1.188	0.693
D13	1.001	0.997	2.360	0.054	1.147	0.729	3.129	0.039	1.188	0.693

Abbreviations: D, day; RR, risk ratio.

### Subgroup analysis

**FIGURE 3:**
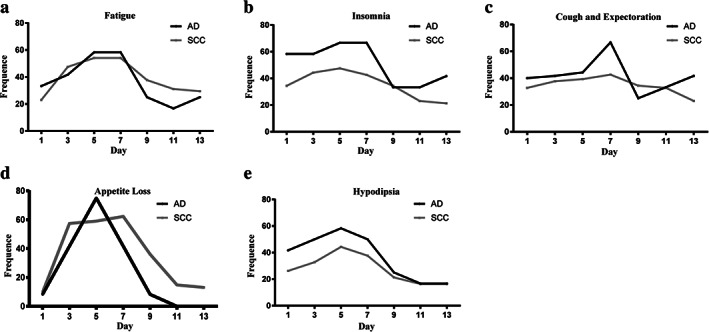
The time‐based symptom appearance rate of the top five most common symptoms in patients with different pathology: Fatigue, insomnia, cough/sputum, appetite loss and hypodipsia. AD, adenocarcinoma; SCC, squamous cell carcinoma

**FIGURE 4:**
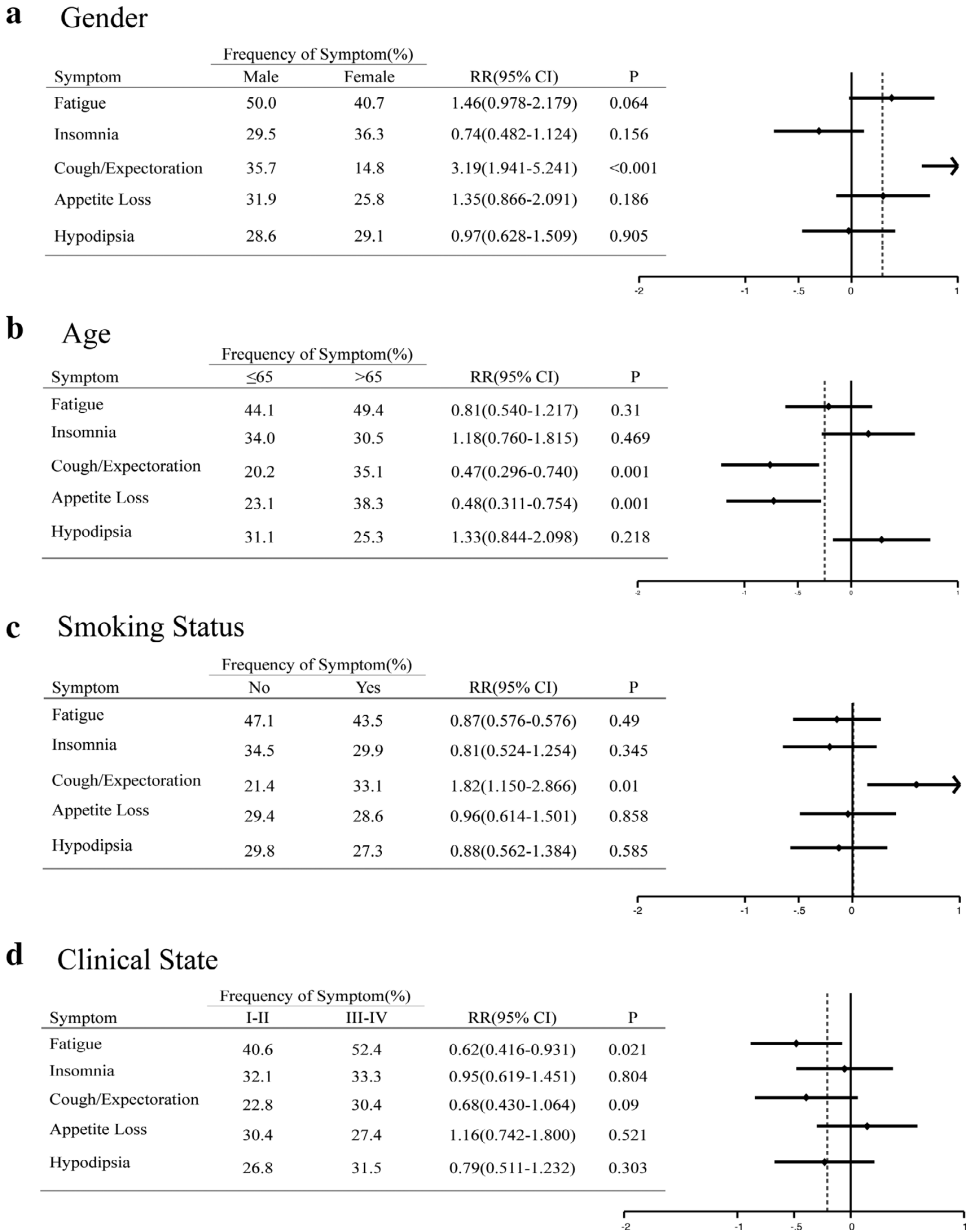
Forest plot of the five most common adverse effects in treatment group in which there were patients of different ages, gender, smoking status and clinical state. CI, confidence interval; RR, relative risk

**FIGURE 5:**
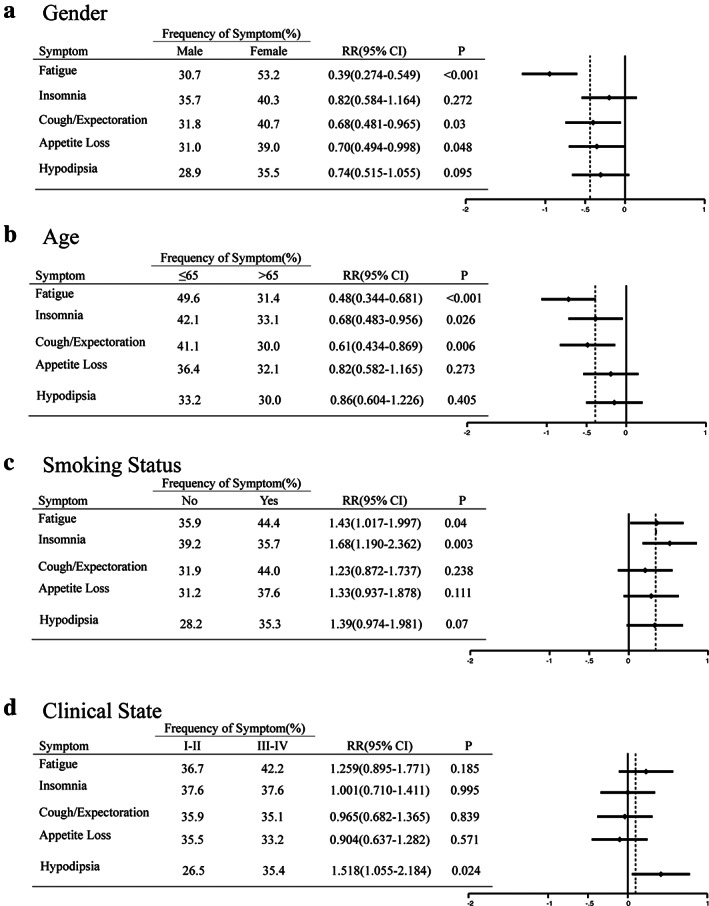
Forest plot of the five most common adverse effects in the control group in which there were patients of different ages, gender, smoking status and clinical state. CI, confidence interval; RR, relative risk

Figures 3, 4, 5 and Table 7 represent subgroup analyses which were performed in order to explore whether there were obvious differences in AEs of chemotherapy and sensitivity to intervention in NSCLC patients with different pathology, gender, age, smoking status and clinical stages.

**TABLE 7:** The difference of 18 symptoms in NSCLC patients with adenocarcinoma and squamous cell carcinoma

Symptom	AD	SCC	RR	95% CI		*p*
	Frequency (%)	Frequency (%)		Low	Up	
Fatigue	41.2	36.9	1.20	0.739	1.944	0.462
Insomnia	36.3	51.2	0.54	0.339	0.870	0.010
Cough/sputum	36.8	42.9	0.78	0.482	1.246	0.293
Appetite loss	37.2	25.0	1.78	1.046	3.028	0.032
Hypodipsia	30.9	39.3	0.69	0.426	1.122	0.134
Anorexia	29.7	11.9	3.13	1.568	6.259	0.001
Dyspnea	31.1	1.2	37.55	5.172	272.586	<0.001
Nausea/vomiting	27.2	15.5	2.04	1.087	3.819	0.024
Ventosity	20.6	38.1	0.42	0.256	0.695	0.001
Perspiring	21.3	16.7	1.35	0.729	2.514	0.335
Depression	18.5	13.1	1.51	0.764	2.972	0.234
Headache/dizziness	17.6	9.5	2.02	0.937	4.372	0.068
Palpitate	17.3	4.8	4.19	1.489	11.802	0.003
Hiccups	7.7	9.5	0.80	0.354	1.789	0.580
Pruritus	6.3	4.8	1.35	0.460	3.964	0.584
Edema	3.3	0.0	0.97	0.950	0.984	0.188
Numbness	1.4	0.0	0.99	0.975	0.997	0.590
Fever	2.1	0.0	0.98	0.965	0.993	0.374

Abbreviations: AD, adenocarcinoma; CI, confidence interval; RR, risk ratio; SCC, squamous cell carcinoma.

Due to the different therapeutic regimens for adenocarcinoma and squamous cell carcinoma, Figure 3 and Table 7 show the relevant subgroup analysis. Although some patients with squamous cell carcinomas may have been treated with gemcitabine on the eighth day, the two different groups had similar development tendency as can be seen in the chart. From Table 7 we can see that there are no obvious difference in most symptoms between different pathology, such as fatigue (RR, 1.20; 95% CI: 0.739–1.944; *p* = 0.462), cough/sputum (RR, 0.78; 95% CI: 0.482–1.246; *p* = 0.293) and hypodipsia (RR, 0.69; 95% CI: 0.426–1.122; *p* = 0.134). However, in the top five most common symptoms, NSCLC patients diagnosed with squamous cell carcinoma were more likely to suffer from insomnia (*p* = 0.01) and patients with adenocarcinoma to suffer from appetite loss more easily (*p* = 0.032).

Female patients tended to have a higher incidence of fatigue (RR, 0.39; 95% CI: 0.274–0.549; *p* < 0.001) and cough/expectoration (RR, 0.68; 95% CI: 0.481–0.965; *p* = 0.03), and no significant differences in insomnia, hypodipsia and appetite loss were observed between male and female patients (Figure 4). What is different from routine cognition is that younger patients (≤65 years old) were more likely to suffer from fatigue (RR, 0.48; 95% CI: 0.344–0.681; *p* < 0.001), cough/expectoration (RR, 0.61; 95% CI: 0.434–0.869; *p* = 0.006) and insomnia (RR, 0.68; 95% CI: 0.483–0.956; *p* = 0.026) after chemotherapy. Compared with patients who had no previous smoking history, smokers were more vulnerable to certain side effects such as fatigue (RR, 1.43; 95% CI: 1.017–1.997; *p* = 0.04) and insomnia (RR, 1.68; 95% CI: 1.19–2.362; *p* = 0.003). Patients with advanced lung cancer (stage III to IV) were found to be more vulnerable to hypodipsia (RR, 1.52; 95% CI: 1.055–2.184; *p* = 0.024).

On the other hand, the sensitivities to low dosage dexamethasone in different lung cancer populations were slightly different, as shown in Figure 5. Compared to male cases, female patients were able to obtain more obvious relief in cough/expectoration (RR, 3.189; 95% CI: 1.941–5.241; *p* < 0.001) after treatment with dexamethasone was prolonged. The intervention treatment significantly eased symptom burdens of cough/expectoration (RR, 0.47; 95% CI: 0.296–0.74; *p* = 0.001) and appetite loss (RR, 0.48; 95% CI: 0.311–0.754; *p* = 0.001) in younger patients (≤65 years old). A meaningful difference was observed in symptom burden of cough/expectoration (RR, 1.82; 95% CI: 1.15–2.866; *p* = 0.01) in non‐smokers. Early‐stage patients (stage I to II) were inclined to reap greater benefits in the side effects of fatigue (RR, 0.62; 95% CI: 0.416–0.931; *p* = 0.021), but there was no obvious difference seen in patients with the remaining four symptoms.

## DISCUSSION

This study explored symptom burden in patients and its influence on the health‐related QOL of lung cancer patients who received standard chemotherapy during a special post‐chemotherapy period. The results provide a significant insight into the benefits of prolonging treatment with dexamethasone to alleviate patients' symptom burden, promote rapid recovery after chemotherapy and improve their QOL. Currently lung cancer, mainly NSCLC, is ranked the highest of all cancer‐related deaths. In recent years, a topical research focus has been on the development of new antitumor drugs. Molecularly targeted therapy and immunotherapy have made considerable headway in antitumor treatment. Nevertheless, there is no doubt that conventional chemotherapy remains a cornerstone in the management of patients with NSCLC, due to patients being offered treatment with molecularly targeted therapy and immune checkpoint inhibitors. However, it is regrettable that research on alleviating chemotherapy‐related AEs is still rather limited. The advantages of this study are that we explored the development tendency of symptoms over time and gathered symptom clusters in order to explore their influence on HRQOL. In addition, this is the first study to explore how to reduce the burden of side effects in patients via prolonging the administration of low dose dexamethasone.

NSCLC patients undergoing chemotherapy often suffer several adverse effects. According to the frequency of occurrence in this study of about 18 items in the symptom diary during a chemotherapy treatment cycle, we identified the following five most common side effects: fatigue (48.5%; M, 33.5; SD, 19.2), insomnia (38.7%; M, 35.0; SD, 33.0), cough and sputum (37.8%), appetite loss (35.2%; M, 21.1; SD, 29.2) and hypodipsia (32.3%). All these symptoms are usually not serious and life‐threatening, but it is precisely these “unimpressive” symptoms which will prejudice patients' physical and psychological health and reduce their QOL. Our research results are in good agreement with a previous similar study of lung cancer patients in Taiwan which also identified five of the most severe symptoms: fatigue (6.68 ± 3.04), sleep disturbance (6.04 ± 3.29), lack of appetite (5.83 ± 3.33), shortness of breath (5.59 ± 3.43), and distress (5.27 ± 3.11).^3^ Analogously, another trial which studied a sample of 249 Chinese patients also came to a similar conclusion.^18^ Taking the conclusions of these trials into consideration we found that fatigue and insomnia were the most common symptoms in NSCLC patients undergoing treatment with chemotherapy.^19^ Broeckle et al.^8^ found that patients with insomnia often suffered more severe fatigue which supported our symptom cluster findings. For example, fatigue and insomnia did not exist independently but interfered with each other, thereby setting up a vicious circle.

In this study, we also performed a factor analysis and a hierarchical cluster analysis. Finally we filtered three common factors and symptom clusters: hypodipsia, anorexia, appetite loss, abdominal distension and nausea/vomiting were gathered as a gastrointestinal symptom cluster; fatigue, insomnia and depression were gathered as a fatigue‐insomnia‐emotion cluster; and cough/expectoration and chest tightness were gathered as a respiratory cluster. Interestingly, our results are similar to the symptom clusters pattern found in other trials of patients with different tumors in different countries.^18^, ^20^, ^21^, ^22^ Therefore, we think it is plausible to suggest that the symptom cluster pattern and its mechanism in patients undergoing chemotherapy are not associated with the type of cancer.

It is not just the side effects in a symptom cluster that can interact with each other. Hsu et al.^13^ reported a fatigue‐anorexia cluster in which fatigue was found to be closely associated with hypodipsia and gastrointestinal symptoms. The secretion of proinflammatory cytokines can diminish appetite by inhibiting the production of cortisol and increase fatigue via reducing the production of adenosine triphosphate (ATP).^23^ Furthermore, loss of appetite resulted in reduced water intake further aggravating hypodipsia.^24^ The report of Farhangfar et al. indicated that hypodipsia and fatigue have a significant correlation with dietary intake, and that these side effects may result in dehydration, reduction in circulation volume and contribute to heavy fatigue.^25^ In addition, emotion‐nausea cluster has been proposed in the study of Hsu et al.^13^ in which nausea was considered to influence insomnia, fatigue and emotion in patients. This is possibly because nausea and vomiting involve higher‐order brain areas associated with emotion control, so that nausea may result in emotional distress.^26^ Emotional distress and nausea may lead to pain and sleep disorders. Insomnia might increase the severity of other symptoms such as nausea and sadness.

Currently glucocorticoids are widely used in the pretreatment of patients who are undergoing chemotherapy. Patients are often administered dexamethasone on the day before chemotherapy and during the treatment period to alleviate symptom burden. A few trials have suggested that 4 mg dexamethasone daily can effectively improve fatigue.^27^ The function of dexamethasone in the prevention and treatment of appetite loss is quick and highly effective but is short in duration.^28^ Cough and dyspnea are the most common symptom burden for patients and dexamethasone has been found to have some efficacy in preventing or relieving cough.^29^, ^30^ The NCCN guide also highlights that dexamethasone can be used in patients with nausea, insomnia and fatigue to ease their symptom burden. Clinically, dexamethasone is often used to prevent and treat chemotherapy‐related side effects. However, there have been no systematic trials providing clinical evidence to prove the function of dexamethasone in alleviating symptom burden during a special post‐chemotherapy period. After patients had received a week of low dose dexamethasone treatment (5 mg/QD), we observed that the three most common side effects of treatment group had achieved significant remission compared with the control group: fatigue (RR, 1.48; 95% CI: 1.120–1.961; *p* = 0.006), insomnia (RR, 1.34; 95% CI: 1.016–1.778; *p* = 0.038) and cough and expectoration (RR, 2.00; 95% CI: 1.484–2.695; *p* < 0.001). However, no significant difference in appetite loss (*p* = 0.095) and hypodipsia (*p* = 0.270) were observed. Patients' health functioning scales were enhanced remarkably within positive effect sizes. The point that low dose dexamethasone could remarkably ease the symptom burden caused by chemotherapy and improve HRQOL of patients was verified by our trial results.

In addition, in the subgroup analysis we found that female patients appeared to suffer more from the side effects of fatigue and cough/expectoration. Younger patients (≤65 years old) were more likely to suffer from fatigue, insomnia and cough/expectoration,contrary to previous findings.^9^ Both younger and female patients also benefited more easily from the intervention. Patients who had a previous smoking history were more likely to suffer from fatigue and insomnia and smoking status made the alleviation of symptom burden appear more difficult. Patients without a smoking status is a good prognostic factor. Early‐stage patients (stage I to II) were more inclined to reap more benefits with regard to the side effects of fatigue.

These preliminary findings have the following implications in clinical practice: (i) the most common side effects found in this study means that medical workers can take corresponding pretreatment measures in advance to alleviate the symptom burden of patients after chemotherapy as much as possible; (ii) healthcare providers should pay more attention to symptom clusters and adopt targeted intervention measures for symptom management by identifying patients who have similar symptom clusters; (iii) the use of dexamethasone has obtained significant results and further research to explore suitable dose rates and more suitable intervention measures are required; and (iv) the results of subgroup analysis on age, gender, smoking status and clinical state are of great significance for targeted intervention measures to be adopted for different patient population. However, there are several limitations in this trial. First, our study failed to strictly limit the chemotherapy cycle of recruited NSCLC patients. The interference of chemotherapy cycle on the study results cannot be ruled out and further studies are needed to monitor symptom clusters in every chemotherapy cycle. Second, the data from the symptom diary which were only qualitative reflects whether side effects occur or not but not the severity of side effects and its variation tendency during chemotherapy. Next, our results were recruited from a single cancer center which limited its potential generalizability to larger‐sample NSCLC patients of different regions and different races. Finally, the study failed to explore the connection between chemotherapy‐related symptom clusters and its underlying biological mechanism.

In conclusion, the study revealed several side effects which caused serious problems to HRQOL of patients with chemotherapy and explored the symptom cluster pattern during chemotherapy. Healthcare providers can take preventive measures in advance according to the variation tendency of side effects to improve the nursing efficiency of patients undergoing chemotherapy. The results of subgroup analysis also provide a basis for us to provide personalized symptomatic treatment measures for patients of different genders, ages and clinical stages. Significantly, the dexamethasone was well tolerated in NSCLC patients who received platinum‐based chemotherapy and substantially alleviated the symptom burden and improved the HRQOL of patients. In general, the results of this study can help physicians to improve their understanding of variation tendency of chemotherapy‐related side effects, develop reference guidelines for pretreatment of these symptoms, promote enhanced recovery after chemotherapy more effectively and improve the HRQOL of patients.

## CONFLICT OF INTEREST

The authors declare that they have no competing interests.
